# Neurodevelopmental Outcome after Culture-Proven or So-Called Culture-Negative Sepsis in Preterm Infants

**DOI:** 10.3390/jcm13041140

**Published:** 2024-02-17

**Authors:** Luca Bedetti, Lucia Corso, Francesca Miselli, Isotta Guidotti, Carlotta Toffoli, Rossella Miglio, Maria Federica Roversi, Elisa della Casa Muttini, Marisa Pugliese, Natascia Bertoncelli, Tommaso Zini, Sofia Mazzotti, Licia Lugli, Laura Lucaccioni, Alberto Berardi

**Affiliations:** 1 Neonatal Intensive Care Unit, University Hospital of Modena, 41124 Modena, Italy; miselli.fnc@gmail.com (F.M.); guidotti.isotta@aou.mo.it (I.G.); roversi.federica@aou.mo.it (M.F.R.); dellacasa.elisa@aou.mo.it (E.d.C.M.); marisa.pugliese@unimore.it (M.P.); natascia.bertoncelli@gmail.com (N.B.); tommaso.zini@yahoo.it (T.Z.); lugli.licia@aou.mo.it (L.L.); 2 Pediatric Postgraduate School, University of Modena and Reggio Emilia, 41121 Modena, Italy; lucia5corso@gmail.com (L.C.); carlotta.toffoli@gmail.com (C.T.); sofia.mazzotti95@gmail.com (S.M.); 3 PhD Program in Clinical and Experimental Medicine, University of Modena and Reggio Emilia, 41124 Modena, Italy; 4 Department of Statistical Sciences, University of Bologna, 41121 Bologna, Italy; rossella.miglio@gmail.com; 5 Pediatric Unit, Department of Medical and Surgical Sciences of the Mother, Children and Adults, University of Modena and Reggio Emilia, 41121 Modena, Italy; laura.lucaccioni@unimore.it

**Keywords:** newborn, very low birth weight, sepsis, culture-proven sepsis, culture-negative sepsis, neurodevelopmental outcome, neurodevelopmental follow-up, functional disability, cerebral palsy

## Abstract

(1) Background: Prematurity is a serious condition associated with long-term neurological disability. This study aimed to compare the neurodevelopmental outcomes of preterm neonates with or without sepsis. (2) Methods: This single-center retrospective case–control study included infants with birth weight < 1500 g and/or gestational age ≤ 30 weeks. Short-term outcomes, brain MRI findings, and severe functional disability (SFD) at age 24 months were compared between infants with culture-proven or culture-negative sepsis or without sepsis. A chi-squared test or Mann–Whitney U test was used to compare the clinical and instrumental characteristics and the outcomes between cases and controls. (3) Results: Infants with sepsis (all sepsis n = 76; of which culture-proven n = 33 and culture-negative n = 43) were matched with infants without sepsis (n = 76). Compared with infants without sepsis, both all sepsis and culture-proven sepsis were associated with SFD. In multivariate logistic regression analysis, SFD was associated with intraventricular hemorrhage (OR 4.7, CI 1.7–13.1, *p* = 0.002) and all sepsis (OR 3.68, CI 1.2–11.2, *p* = 0.021). (4) Conclusions: All sepsis and culture-proven sepsis were associated with SFD. Compared with infants without sepsis, culture-negative sepsis was not associated with an increased risk of SFD. Given the association between poor outcomes and culture-proven sepsis, its prevention in the neonatal intensive care unit is a priority.

## 1. Introduction

Premature birth is commonly associated with significant complications, including bronchopulmonary dysplasia, intraventricular hemorrhage, necrotizing enterocolitis, patent ductus arteriosus, and retinopathy of prematurity. It is widely recognized that the severity of complications increases with decreasing gestational age and birth weight, leading to higher rates of mortality and long-term neurodevelopmental impairment in these infants [1,2,3,4]. Among the complications associated with prematurity, there is also an increased susceptibility to experiencing invasive infections such as sepsis and meningitis. This susceptibility is attributable to multiple factors such as the immaturity of the immune systems, the reduced thickness and efficacy of the cutaneous barrier, the need to undergo invasive medical interventions (i.e., catheterization and mechanical ventilation), and the need for a prolonged hospital stay [5,6,7]. Despite therapeutic and hygienic advancements, sepsis remains a primary cause of morbidity and mortality in neonates, particularly in very-low-birth-weight (VLBW) infants. As a matter of fact, compared with full-term, the risks of sepsis and related mortality in VLBW neonates may be more than 50 and 400 times higher, respectively [7]. 

Sepsis represents a relevant issue as it is increasingly evident that it has a significant role in the pathogenesis of cerebral damage in neonates, through both direct and indirect mechanisms [8]. Direct mechanisms include the disruption of the blood–brain barrier (due to direct exposure to bacterial cell wall components and inflammatory cytokines) or the entry of cytokines into the central nervous system [8]. In the context of indirect mechanisms, cardiovascular instability associated with septic shock, cerebral hypoperfusion, and cerebral hypoxia–ischemia all play relevant roles; indeed, the capacity for the autoregulation of cerebral blood flow is impaired in preterm infants, and it is further compromised during inflammation [9]. These events may damage the vulnerable white matter, primarily manifesting as cystic periventricular leukomalacia or non-cystic diffuse white matter injury [10]. In addition to these lesions, which are easily detectable through neuroimaging, stress-inducing factors such as inflammation, together with the interventions required to treat sepsis (i.e., mechanical ventilation or catecholamine support), can also lead to more subtle changes (such as dysmaturation) that may not be detectable as early in the process through neuroimaging but may have potential negative effects on long-term neurodevelopmental outcomes [11].

While these findings ideally support an association between sepsis and potential long-term neurological impairment, only a few studies have investigated the effect of this association [9,11]. Bacterial sepsis is frequently categorized based on its timing of onset (early or late onset) and culture status (positive or negative). Both early- and late-onset sepsis are known to be variably associated with neurodevelopmental impairment. In a study that investigated the 5-year neurodevelopmental outcome among very preterm infected infants, early-onset sepsis was associated with severe intraventricular hemorrhage and an increased relative risk of death, neurodevelopmental impairment at 2 years of age, and cerebral palsy [12]. Similarly, late-onset sepsis was found to be associated with a higher risk of the combined outcome of death and neurodevelopmental impairment, and of death alone [13]. However, studies in this field differ in terms of their definition of the neurological outcomes, and the characteristics of the populations may vary (for example, gestational age, number of episodes, or rates of meningitis complicating sepsis) [14].

Furthermore, an important question concerns infants with so-called culture-negative sepsis (CNS), a controversial condition whose actual existence is much debated [15]. Indeed, some infants exhibit sterile cultures, nonspecific symptoms, and abnormal laboratory markers. They are frequently treated with empirical antibiotics and are therefore diagnosed as having CNS. We do not know how many infants diagnosed with CNS actually suffer from conditions other than bacterial sepsis (i.e., viral infections, noninfectious systemic inflammatory response, or focal bacterial infection with negative blood culture), given the current diagnostic difficulty in establishing the infectious nature of the disease with certainty. However, a recent editorial has emphasized the likely extreme rarity of sepsis in most of these infants [15]. For these reasons, clinicians are called upon to seek clarity on this issue, given its clinical implications and the relatively high number of infants suffering from sepsis and meningitis.

The knowledge acquired so far suggests the relevance of assessing neurodevelopmental impairment in survivors of neonatal sepsis or meningitis. This is important because the early identification of an impairment and the institution of appropriate interventions has been shown to improve the outcomes of affected infants, including motor and cognitive outcomes, as well as hearing outcomes. The American Academy of Pediatrics, United Kingdom National Institute for Health and Care Excellence, and European Standards of Care for Newborn Health recommend screening with validated tools at 9, 18–24, and 30–36 months [16,17].

For the reasons outlined above, clinicians are called upon to seek clarity on this issue, given its clinical implications and the relatively high number of infants suffering from sepsis and meningitis.

The aim of this study was to compare the neurodevelopmental outcomes of preterm neonates with or without sepsis at age 24 months. Both culture-proven sepsis (CPS) and so-called CNS were included.

## 2. Materials and Methods

### 2.1. Study Design

This is a single-center retrospective case–control study conducted on a cohort of newborns admitted to the Neonatal Intensive Care Unit (NICU) of Polyclinic University Hospital of Modena (Italy) from 1 December 2009 to 31 December 2016.

Inclusion criteria were CPS or CNS during the hospital stay, VLBW (birth weight < 1500 g), and/or gestational age under 30 weeks. Controls were premature infants with comparable gestational age and birth weight without CPS/CNS. Exclusion criteria were major congenital malformations, chromosomal abnormalities, genetic or metabolic disorders, congenital heart diseases, death before hospital discharge, or transfer to other medical facilities.

### 2.2. Definitions

Sepsis: life-threatening organ dysfunction caused by a dysregulated host response to infection [18,19,20,21,22,23].
Early-onset sepsis: onset of symptoms and isolation of pathogens from blood and/or cerebrospinal fluid within the first 72 h of life [19,20,23].
Late-onset sepsis: onset of symptoms and isolation of pathogens from blood and/or cerebrospinal fluid after the first 72 h of life [19,20,23].
CPS: isolation of a pathogen from normally sterile sites (blood, cerebrospinal fluid, or other sterile sites) in the presence of typical symptoms of sepsis [19,20,21].
CNS: clinical and/or laboratory signs of sepsis with sterile blood/cerebrospinal fluid culture in an infant who has received antibiotic treatment for at least 5 days [19,20,24].

### 2.3. Data Collection

Clinical and instrumental data pertaining to each individual case were retrospectively extracted from both neonatal hospitalization and outpatient neurological follow-up records.

For each patient, we fulfilled a comprehensive anonymous set of data encompassing demographic and clinical characteristics at birth (sex, gestational age, body weight, twinning, mode of delivery, use of prenatal steroids, Apgar score, and chorioamnionitis), during hospital stay (total days of central line, surfactant administration, use of postnatal steroids, patent ductus arteriosus and treatment [25], diagnosis of bronchopulmonary dysplasia [26], necrotizing enterocolitis [23], retinopathy of prematurity [27], and brain lesions), and at 24 months of corrected age (neurodevelopmental outcome). Comprehensive information pertaining to sepsis episodes was documented and categorized into distinct classifications as follows: early-onset sepsis, late-onset sepsis, CPS, and CNS.

### 2.4. Neurodevelopmental Assessment at 24 Months of Age

Data concerning neurodevelopment were procured via sequential evaluations of psychomotor development, extending until a minimum age of 24 months, corrected for prematurity. The neurodevelopmental assessment was carried out via strict adherence to a standardized protocol for high-risk newborns developed in our NICU. A developmental psychologist, a child physiotherapist, and a neonatologist were dedicated to neurodevelopmental assessment. To ensure adherence, parents received timely telephone reminders of upcoming appointments.

The standardized neurodevelopmental examination was composed by Amiel-Tison and Grenier with an extension of Touwen’s method [28], using the Griffiths Mental Development Scale [29]. The Griffiths Mental Development Scale (0–2 years) provides a general development quotient of infants’ abilities, with a mean of 100.5, an SD of 15, and 5 subscale quotients (locomotor, eye and hand coordination, personal and social, hearing and language, and cognitive performance).

The global outcome at 24 months of corrected age was classified into the following categories: normal (no evidence of neurological impairment and general development quotient >85 on the Griffiths scale), mildly abnormal (presence of clumsiness and/or balance difficulties and general development quotient between 70 and 85 on the Griffiths scale), and severely abnormal (evidence of cerebral palsy, bilateral blindness, bilateral deafness, and/or general development quotient <70). When at least one of the assessments was severely impaired, the presence of a severe functional disability (SFD) was stated.

### 2.5. Brain MRI Evaluations

Following the established internal protocol within our neuroradiology unit, a systematic approach was taken to conduct brain MRI examinations. The MRI assessment was performed within 37 and 44 weeks of gestational age. The examination was conducted under pharmacological sedation using a 1.5-T scanner (Intera; Philips Medical Systems, Best, The Netherlands). During these MRI sessions, a comprehensive set of both conventional and diffusion-weighted MRI sequences was acquired. These sequences were subsequently interpreted by two seasoned operators possessing substantial experience in this domain. To ensure the utmost accuracy and relevance of our assessments, we adhered to the classification framework introduced by Arulkumaran and colleagues [30], which offers a contemporary and comprehensive approach to categorizing these identified conditions. Image examinations include assessments for:

- Germinal matrix hemorrhage–intraventricular hemorrhage: The germinal matrix is a structure that is normally visible on imaging and undergoes involution as the fetus ages, leaving only remnants in the caudothalamic notch and roof of the temporal horns after 32 weeks of gestation. An irregular contour in a subependymal area with a low T2 signal or accompanying intraventricular hemorrhage is categorized as germinal matrix hemorrhage–intraventricular hemorrhage [30].
- Hemorrhagic parenchymal infarction: When fully developed, hemorrhagic parenchymal infarction is characterized by a focal bulging or outpouching of the ventricular contour, usually unilateral. This is often accompanied by a low T2 signal component, indicating a previous hemorrhage [30].
- Periventricular leukomalacia: Periventricular leukomalacia typically manifests as multiple bilateral periventricular cysts in a symmetric distribution, initially appearing to be separate from the ventricle. Solitary or unilateral cysts are more likely to be venous infarcts or conatal cysts. Criteria to diagnose periventricular leukomalacia are as follows: residual bilateral periventricular cysts, dilated/angulated posterior aspects of the lateral ventricles, and associated white matter volume loss [30].

### 2.6. Statistical Analysis

The demographic, clinical, and instrumental characteristics of the infants enrolled were subjected to analysis using descriptive statistical methods. Continuous variables were presented as median and interquartile range, whereas categorical variables were depicted as frequencies.

The infants were divided into two groups according to the presence or the absence of sepsis. We selected 76 infants per group using a propensity score analysis with 1:1 matching, using the nearest matching method to minimize the bias stemming from differences in birth weight and gestational age between the two groups. After propensity score matching, we compared the clinical, instrumental, and outcome characteristics between the two groups using a chi-squared test or Mann–Whitney U test, as appropriate. The neurodevelopmental outcomes at the age of two years and their associated factors were evaluated using logistic regression analysis. The odds ratio, serving as a measure of the strength of association, was determined and presented alongside its corresponding 95% confidence interval. A *p* value < 0.05 was considered significant. In relation to a detailed analysis of the scores on the Griffiths Mental Development Scale, infants with cerebral palsy or blindness were excluded. Data were analyzed using STATA software (version 13.0).

### 2.7. Ethical Considerations

The study was conducted in accordance with the Declaration of Helsinki, Edinburgh Revision (2000). The study was approved by the Area Vasta Nord-Emilia Romagna Ethics Committee (protocol number 42/2019). Given the impossibility of retrospectively retrieving the consent of all the infants included in this study, the research ethics committee waived the need for consent.

## 3. Results

**Figure 1:**
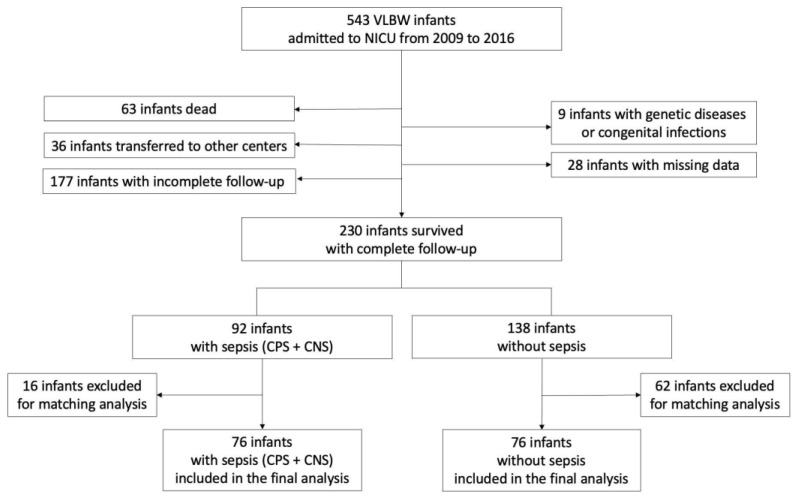
Enrollment flow diagram. CNS—culture-negative sepsis. CPS—culture-proven sepsis. NICU—neonatal intensive care unit. VLBW—very low birth weight.

During the study period, 543 VLBW infants were delivered, of whom 230 completed the 24-month neurodevelopmental follow-up. Of these, 92 (40.0%) had been diagnosed with sepsis (CPS or CNS) and 138 had no sepsis. A total of 76 infants with sepsis (including 33 [43.4%] CPS and 43 [66.6%] CNS) and 76 infants without sepsis (Figure 1) were finally included after matching for body weight and gestational age. Infants with CPS had a significantly lower gestational age (median 27.1 weeks; IQR 26.3–28.6) compared with infants with CNS (median 28 weeks; IQR 27–29.9; *p* < 0.045) and without sepsis (median 29 weeks; IQR 26.6–30; *p* < 0.016).

Two infants had early-onset sepsis (both caused by Gram-negative bacteria (*Enterobacterales*, n = 1; *Escherichia coli*, n = 1)). A total of 31 infants had late-onset sepsis (21 due to Gram-positive bacteria (Group B Streptococcus, n = 4; coagulase-negative Staphylococci, n = 11; *Staphylococcus aureus*, n = 5; others, n = 1); 9 due to Gram-negative bacteria (*Escherichia coli*, n = 4; *Enterobacterales*, n = 3; others, n = 2); 1 due to fungi). Five infants had more than one episode of CPS (three infants had two episodes; two infants had three episodes). Among infants with CPS, five (15.2%) also had meningitis. 

### 3.1. All Sepsis (CPS + CNS) vs. No Sepsis

Compared with infants without sepsis, those with CPS/CNS had lower rates of cesarean section (Table 1). Furthermore, they were more likely to undergo mechanical ventilation (together with a prolonged duration), more likely to have more days with a central line in place, and more likely to have higher rates of medical (or surgical) treatment for PDA or necrotizing enterocolitis; they were more likely to be given postnatal steroids, more likely to have severe bronchopulmonary dysplasia, more likely to require a prolonged hospital stay, and usually older at discharge from hospital (Table 2).

Finally (Table 3), they were more likely to have periventricular leukomalacia and a higher incidence of long-term sequelae (including cerebral palsy). A total of 19 infants with sepsis and 5 infants without sepsis had SFD (details regarding the type of SFD are reported in the footnotes of Table 3). GMDS-R infants with sepsis had significantly lower scores in the B scale.

### 3.2. Culture-Proven Sepsis vs. No Sepsis

Neonates with CPS had lower rates of cesarean section, a lower 5th-minute Apgar score, and a lower gestational age (Table 1). Furthermore, they were more likely to undergo mechanical ventilation (and a prolonged duration of mechanical ventilation), more likely to have more days of having a central line in place, and more likely to have higher rates of treatment for PDA; they were more likely to be given postnatal steroids, more likely to have severe bronchopulmonary dysplasia, more likely to have grade ≥ 3 retinopathy of prematurity, more likely to have a prolonged hospital stay, and more likely to be older at discharge from hospital (Table 2). They were more likely to present with long-term neurodevelopmental sequelae (Table 3). Regarding the Griffiths Mental Development Scale, infants with CPS had significantly lower scores in the B and general development quotient scales.

### 3.3. Culture-Negative Sepsis vs. No Sepsis

Infants with CNS were more likely to undergo mechanical ventilation (and prolonged duration of mechanical ventilation), and more likely to have more days with the central line in place; they were more likely to have severe bronchopulmonary dysplasia or necrotizing enterocolitis or to have higher rates of PDA treatment. They were also more likely to be given postnatal steroids, more likely to have a prolonged hospital stay, and more likely to be older at discharge from hospital (Table 2). In addition, they had a higher risk of developing periventricular leukomalacia.

### 3.4. Sepsis and Severe Functional Disability: Logistic Regression

Table 4 shows the factors associated with SFD in uni- and multivariate analyses. Following univariate analysis, all sepsis (CPS + CNS), intraventricular hemorrhage, and gestational age were significantly associated with SFD. All sepsis (CPS + CNS) and intraventricular hemorrhage remained associated with SFD upon multivariate analysis. 

## 4. Discussion

This study evaluates the neurodevelopmental outcome at two years of corrected age in premature infants with sepsis compared with infants without sepsis. 

Previous studies show that premature infants have an increased risk of sepsis and long-term neurological impairment, which rises with decreasing gestational age [1,2,3,4,5,6,7]. With respect to full-term neonates, extremely preterm or extremely low-birth-weight neonates have a very high risk of late-onset sepsis and related mortality (>100- and >800-fold higher, respectively) [7]. Long-term cognitive impairment is more frequent, by up to 40 times, in extremely preterm-born neonates compared with healthy full-term infants [3]. It has been reported that neonatal sepsis and meningitis are both associated with an increased risk of death [31], and a limited number of studies have described their association with long-term neurodevelopmental impairment [4,6,10,11,32,33]. However, in most studies, infants with sepsis typically have a lower gestational age compared with unaffected infants, which could potentially skew the results [13,24,34,35]. Additionally, previous studies addressing the impact of neonatal infection on neurodevelopmental outcome have conflicting results (regarding, for example, patient characteristics, the specifics of the illness, and long-term follow-up rates), and their comparison is difficult because of the variable definitions of SFD [14]. To date, only one meta-analysis on this topic has been published [14], and, given the limitations of previous studies, it has only been possible to establish a definite association between sepsis and cerebral palsy, thus the results regarding the association between sepsis and SFD should be interpreted with caution.

We compared infants with and without sepsis with a comparable birth weight and gestational age. Any sepsis (both CPS and CNS) was significantly associated with an increased risk of need for mechanical ventilation, central line, PDA treatment, steroid treatment, prolonged hospitalization, and diagnosis of bronchopulmonary dysplasia. It was also associated with periventricular leukomalacia (15.1 vs. 4.4%) and long-term sequelae, including cerebral palsy (15.8 vs. 3.9%), whereas the risk of SFD increased by almost four times (OR: 3.68).

Preterm neonates with both CPS and CNS showed an increased risk of poor multiple short-term outcomes compared with infants without sepsis. Conversely, SFD was associated with CPS but not CNS. This finding is consistent with Schlapbach et al. [13], who reported that only CPS independently increased the risk of cerebral palsy (OR: 3.23) and neurodevelopmental impairment at 18–24 months’ corrected age (OR 1.60), supporting the belief that CPS is a distinct entity from CNS. CNS could be of a non-bacterial origin in most cases [36]. Such a diagnostic error could have clinical consequences, such as inadequate treatment of the true underlying non-bacterial diseases and dysbiosis resulting from unnecessary antibiotics [35]. However, in the literature, the neurodevelopmental outcome of infants with CPS and CNS is less clear and sometimes reported as similar [24,37]. Mukhopadhyay et al. found an increased risk of death and SFD, death alone, or brain injury in infants with CPS, while surviving infants with CNS had only a higher risk of SFD [35]. It should be noted that the currently available studies differ in their definition of SFD; moreover, they do not always correlate the etiology of sepsis (and eventual antibiotic resistance) with the child’s neurological outcome [14]. They rarely consider the severity of the disease and never consider the timeliness and effectiveness of the treatment that was administered, an important aspect that is difficult to assess in studies [32].

The association between sepsis and poor neurodevelopmental outcomes compels us to seek strategies to prevent sepsis in the NICU [38]. In our study, 94% of sepsis cases were late-onset sepsis. Therefore, protocols related to the proper use of central venous catheters, minimizing their duration, and improving total enteral feeding are of the utmost importance [39,40,41]. Bloodstream infection associated with central catheters (CLABSI) is a hospital-acquired and preventable condition that has received particular attention in the last decade. Various quality improvement efforts have been implemented, resulting in the successful reduction in CLABSI in NICUs. Common strategies include a shift in the mindset regarding CLABSI from inevitable to preventable targeted commitment to the formal training of NICU staff in quality improvement principles, and the development and implementation of bundles for central catheter insertion and maintenance based on best-practice recommendations, the formation of dedicated central catheter teams, the establishment of collaborations for CLABSI prevention, and the reporting and transparency of data. These strategies are extremely important and deserve the widest possible dissemination as they have demonstrated effectiveness in infants of all gestational ages [40].

Also, minimizing the use of unnecessary antibiotics [42] is a well-known factor that can reduce sepsis during NICU stays [43,44,45,46]. There is a strong association between the prolongation of empirical antibiotic courses in the first days of life and the risk of subsequent late-onset sepsis [43,45]. Finally, an early diagnosis and appropriate treatment of sepsis, especially septic shock, are of paramount importance in limiting cerebral lesions due to infections [47,48]. The outcome of septic shock is well-known to depend on the prompt identification of symptoms suggestive of sepsis and strict adherence to cardio-pulmonary resuscitation guidelines, protocols, and algorithms. Fluid administration, coupled with the immediate and aggressive use of bactericidal antibiotics, plays a fundamental role. A delay in initiating antibiotic therapy has been associated with poor outcomes and an increased risk of mortality. Therefore, the use of antimicrobials is recommended as soon as possible, ideally within 1 h of diagnosis, following the “1 h sepsis bundle”, which includes collecting blood cultures before antibiotic administration, administering broad-spectrum antibiotics, and completing a crystalloid fluid bolus. In cases of septic shock, the antibiotics that are used should be broad-spectrum and effective against the main pathogens of early-onset sepsis (with a combination of ampicillin and gentamicin) and late-onset sepsis (with a combination of oxacillin or nafcillin and an aminoglycoside). Third-generation cephalosporins should be added when meningitis is suspected. Lumbar puncture remains an essential tool for diagnosing meningitis and further guiding appropriate antibiotic therapies [49].

In addition, the economic costs of sepsis and its consequences must be assessed. Prematurity itself is associated with higher hospital costs compared with full-term birth [50], and an Italian study found that prematurity without prematurity-related morbidities was associated with higher costs, not only during hospital stay but also after home discharge (in this study, the follow-up lasted until 18 months of life) [51]. The costs of preterm birth following the initial hospitalization go beyond those of the healthcare system, particularly for infants developing disabilities. This includes the need for special education assistance and direct economic losses to the family members of newborns (such as reduced earnings and losses resulting from paying uncovered drugs or travel costs) [51,52]. Neonatal sepsis is an additional factor contributing to the increase in the cost of care of preterm infants [53], both complicating hospital care and being associated with future disabilities. In our study, this latter aspect becomes evident. Sepsis was associated with an increase in the need for interventions (longer duration of central catheter use, increased need for postnatal steroids, mechanical ventilation, and treatment of PDA), an increase in the duration of hospital stay, and an increase in long-term neurodevelopmental sequelae. All these aspects are associated with increased costs.

The strengths of our study include its exhaustive definition of SFD and the recentness of the cases included. This last aspect is particularly relevant because the evaluated patients are those who benefited from the most advanced treatments currently available to us.

This study has several limitations. First, an important potential limitation concerns the burden of actually infected infants among those with CNS, since it is currently difficult to determine with certainty how many of them actually suffered from bacterial infection other than sepsis. In addition, the sample size is limited. This had various implications for the type of statistical analysis we could conduct; furthermore, compared with larger studies, small studies more frequently present a higher risk for SFD in infants with sepsis [14]. Even though we matched infants for birth weight and gestational age, the group of infants with CPS differed significantly in gestational age (but not in birth weight) from the groups of infants with CNS or without sepsis, having a lower gestational age. This difference could represent a potential bias in the assessment of the association between CPS and neurodevelopmental outcomes at 2 years of corrected age in comparison with the other groups. Finally, the follow-up concluded at 2 years of age, limiting the assessment of minor developmental impairments diagnosed at a later age.

## 5. Conclusions

This study evaluated the neurodevelopmental outcome at two years of corrected age in premature infants with sepsis compared with infants without sepsis and the differences between infants with CPS or CNS. Both forms of sepsis (CPS or CNS) were associated with SFD. However, when these were evaluated separately, only CPS was associated with an increased risk of SFD. This study reinforces the hypothesis that CPS is a risk factor for long-term neurological impairment and underlines that its prevention in the NICU is a priority. Clinicians and researchers have the decisive role to implement measures to minimize the risk of sepsis in neonates admitted to the NICU and to treat sepsis as soon as possible and in the best way. Further studies should assess the additional characteristics of neonates with CPS or CNS, including data on clinical stability during hospital stays.

## Figures and Tables

**Table 1:** Perinatal characteristics of infants and comparison between different categories of infants based on sepsis.

Variables	All Sepsis (CPS + CNS)	*p* ^†^	CPS	*p* ^§^	CNS	*p* ^¥^	Infants without Sepsis
	n = 76		n = 33		n = 43		n = 76
Sex, male	38 (50)	0.871	17 (51.2)	0.985	21 (48.8)	0.795	39 (51.3)
Gestational age	27.7 (26.8; 29.6)	0.139	27.1 (26.3; 28.6)	0.016	28 (27; 29.9)	0.842	29 (26.6; 30)
Birth weight	959 (805; 1200)	0.575	960 (773; 1210)	0.557	970 (829; 1146)	0.727	955 (820; 1146)
SGA	11 (14.5)	0.656	4 (12.1)	0.510	7 (16.3)	0.908	13 (17.1)
Twinning	28 (36.9)	0.610	15 (45.5)	0.211	13 (30.2)	0.765	25 (32.3)
Cesarean section	55 (72.4)	0.047	21 (63.6)	0.010	34 (79.1)	0.366	65 (83.5)
Prenatal steroids ^¶^	10 (14.1)	0.758	7 (23.3)	0.381	3 (7.3)	0.189	11 (15.9)
5th minute Apgar score	8 (7–9)	0.105	8 (7–8)	0.044	8 (7–9)	0.454	8 (7–9)
Chorioamnionitis	23 (31.6)	0.594	11 (33.3)	0.548	13 (30.2)	0.763	21 (27.6)

CNS—culture-negative sepsis. CPS—culture-proven sepsis. SGA—small for gestational age. Data presented as median (interquartile range) or n (%). Missing data: prenatal steroids, n = 12; 5th Apgar score, n = 2. ^†^ Comparison between infants with sepsis and infants without sepsis. ^§^ Comparison between infants with CPS and infants without sepsis. ^¥^ Comparison between infants with CNS and infants without sepsis. ^¶^ Considered if given at least 48 h prior to delivery.

**Table 2:** Short-term outcomes and comparisons between different categories of infants based on sepsis.

Variables	All Sepsis (CPS + CNS)	*p* ^†^	CPS	*p* ^§^	CNS	*p* ^¥^	Infants without Sepsis
	n = 76		n = 33		n = 43		n = 76
Days of central line	29 (14; 39)	<0.001	33 (22–44)	<0.001	26 (12–36)	0.001	15 (6; 22)
Surfactant	59 (73.6)	0.105	26 (78.8)	0.175	33 (76.7)	0.211	50 (65.8)
Post-natal steroids	15 (19.7)	0.008	8 (24.2)	0.004	7 (16.3)	0.049	4 (5.3)
MV	61 (80.3)	<0.001	30 (90.9)	<0.001	31 (72.1)	0.001	36 (47.4)
MV, days	5 (1; 15)	<0.001	7 (2–10)	<0.001	4 (0–17)	<0.001	0 (0–3)
BPD		<0.001		<0.001		0.003	
- Mild	22 (29.0)		13 (39.4)		9 (20.9)		22 (29.0)
- Moderate	13 (17.1)		5 (15.2)		8 (18.6)		6 (7.9)
- Severe	15 (19.8)		8 (24.2)		7 (16.3)		1 (1.3)
PDA		0.007		0.021		0.031	
- Medical treatment	34 (44.7)		14 (42.4)		20 (46.5)		20 (26.3)
- Surgical treatment	5 (6.6)		3 (9.1)		2 (4.7)		1 (1.3)
NEC ^‖^	4 (5.3)	0.043	1 (3)	0.127	3 (7.0)	0.020	0
ROP ≥ grade 3	5 (6.7)	0.246	4 (12.1)	0.046	1 (2.3)	0.919	2 (2.6)
IVH	18 (24.7)	0.085	9 (29.0)	0.059	9 (21.4)	0.259	9 (13.2)
HPI	6 (8.2)	0.065	3 (9.7)	0.054	3 (7.1)	0.123	1 (1.5)
PVL	11 (15.1)	0.034	4 (12.9)	0.126	7 (16.7)	0.030	3 (4.4)
Hospital stay, days	75 (55; 93)	<0.001	88 (58–100)	<0.001	66 (55–84)	0.013	60 (45; 72)
PCE at discharge	38.6 (36.9; 41)	<0.001	39 (37–41.6)	0.002	38.4 (36.8–40)	0.005	37.2 (35.8; 38.1)

BPD—bronchopulmonary dysplasia. CNS—culture-negative sepsis. CPS—culture-proven sepsis. HPI—hemorrhagic parenchymal infarction. IVH—intraventricular hemorrhage. MV—mechanical ventilation. NEC—necrotizing enterocolitis. PCE—post-natal conceptional age. PDA—patent ductus arteriosus. PVL—periventricular leukomalacia. ROP—retinopathy of prematurity. Data presented as median (interquartile range) or n (%). Missing data: IVH, n = 11. HPI, n = 11. PVL, n = 11. ^†^ Comparison between infants with sepsis and infants without sepsis. ^§^ Comparison between infants with CPS and infants without sepsis. ^¥^ Comparison between infants with CNS and infants without sepsis. ^‖^ Stage ≥ IIA of Bell’s classification [12].

**Table 3:** Neurodevelopmental outcomes and comparisons between different categories of infants based on sepsis.

Variables	All Sepsis (CPS + CNS)	*p* ^†^	CPS	*p* ^§^	CNS	*p* ^¥^	Infants without Sepsis
	n = 76		n = 33		n = 33		n = 76
Neurodevelopmental outcome		0.008		0.004		0.063	
- normal	47 (61.8)		18 (54.6)		29 (67.5)		59 (77.6)
- MFD	10 (13.2)		5 (15.2)		5 (11.6)		12 (15.8)
- SFD	19 (25.0)		10 (30.3) ^‡^		9 (20.9) ^¶^		5 (6.6) *
Motor outcome		0.003		0.007		0.137	
- normal	50 (65.8)		18 (55.6)		32 (74.4)		62 (81.6)
- MFD	14 (18.4)		9 (27.3)		5 (11.6)		11 (14.5)
- SFD	12 (15.8)		6 (18.2)		6 (14.0)		3 (3.9)
GMDS-R ^‖^							
A	98 (98–107)	0.322	98 (98–107)	0.387	98 (98–107)	0.458	98 (98–107)
B	106 (99–115)	0.038	106 (92–106)	0.006	109 (99–119)	0.416	112 (100–119)
C	101 (88–105)	0.621	98 (84–102)	0.179	102 (91–107)	0.719	102 (88–105)
D	107(102–112)	0.762	107 (102–112)	0.259	107 (102–118)	0.644	107 (102–118)
E	101 (99–106)	0.286	100 (99–106)	0.497	102 (100–106)	0.319	106 (100–106)
GQ	103 (96–109)	0.264	100 (95–106)	0.033	105 (97–111)	0.931	105 (98–111)

A—loco-motor scale. B—eye and hand coordination scale. C—personal and social scale. CNS—culture-negative sepsis. CPS—culture-proven sepsis. D—hearing and language scale. E—cognitive performance scale. GMDS-R—Griffiths Mental Development Scale. GQ—general development quotient. MFD—moderate functional disability. SFD—severe functional disability. Data presented as median (interquartile range) or n (%). ^†^ Comparison between infants with sepsis and infants without sepsis. ^§^ Comparison between infants with CPS and infants without sepsis. ^¥^ Comparison between infants with CNS and infants without sepsis. ^‡^ SFD: cerebral palsy, n = 6 (of whom one also had deafness). Blindness, n = 4. ^¶^ SFD: cerebral palsy, n = 7 (of whom one also had blindness). Severe cognitive impairment, n = 2. * SFD: cerebral palsy, n = 3. Blindness, n = 1. Severe cognitive impairment, n = 1. ^‖^ A total of 21 infants with cerebral palsy (n = 16) or blindness (n = 6) were excluded from the analysis. In addition, data were unavailable for 3 infants. Number of infants included in the final analysis were distributed as follows: infants with all sepsis (CPS + CNS), n = 56; infants with CPS, n = 23; infants with CNS, n = 33; infants without sepsis, n = 73.

**Table 4:** Factors associated with severe functional disability in uni- and multivariate analysis.

Variables	Univariate Analysis			Multivariate Analysis		
	OR	CI	*p*	OR	CI	*p*
All sepsis (CPS + CNS)	4.73	1.6–13.4	0.004	3.68	1.2–11.1	0.021
BPD	1.40	0.9–2.1	0.102			
IVH	5.8	2.2–15.4	<0.001	4.7	1.7–13.1	0.002
Gestational age	0.74	0.5–0.9	0.014	0.83	0.6–1.0	0.159
Birth weight	0.99	0.9–1.0	0.071			
Mechanical ventilation	2.43	0.8–6.9	0.096			

## Data Availability

Rough data supporting reported results are made available upon reasonable request to the corresponding author.
